# Economic hardships in adulthood and mental health in Sweden. the Swedish National Public Health Survey 2009

**DOI:** 10.1186/1471-2458-11-788

**Published:** 2011-10-11

**Authors:** Johanna Ahnquist, Sarah P Wamala

**Affiliations:** 1 Department of Public Health Sciences, Division of Applied Public Health, Karolinska Institutet, Stockholm, Sweden; 2 Swedish National Institute of Public Health, Östersund, Sweden

## Abstract

**Background:**

Possible accumulative effects of a combined economic hardship's measure, including both income and non-income related economic hardships measures, on mental health has not been well investigated. The aim of this paper was to investigate; (i) independent associations between multiple measures of economic hardships and mental health problems, and (ii) associations between a combined economic hardships measure and mental health problems.

**Methods:**

We analysed data from the 2009 Swedish National Survey of Public Health comprising a randomly selected representative national sample combined with a randomly selected supplementary sample from four county councils and three municipalities consisting of 23,153 men and 28,261 women aged 16-84 years. Mental health problems included; psychological distress (GHQ-12), severe anxiety and use of antidepressant medication. Economic hardship was measured by a combined economic hardships measure including low household income, inability to meet expenses and lacking cash reserves.

**Results:**

The results from multivariate adjusted (age, country of birth, educational level, occupational status, employment status, family status and long term illness) logistic regression analysis indicate that self-reported current economic difficulties (inability to pay for ordinary bills and lack of cash reserves), were significantly associated with both women's and men's mental health problems (all indicators), while low income was not. In addition, we found a statistically significant graded association between mental health problems and levels of economic hardships.

**Conclusions:**

The findings indicate that indicators of self-reported current economic difficulties seem to be more strongly associated with poor mental health outcomes than the more conventional measure low income. Furthermore, the likelihood of mental health problems differed significantly in a graded fashion in relation to levels of economic hardships.

## Background

There is a growing body of evidence linking mental health to poverty and economic hardships. A majority of previous studies analysing conventional indicators of poverty or economic hardships based on income have found evident associations with poor mental health outcomes such as psychological distress [1-4] and depression [5-10].

Less attention has been paid to other 'non income related' dimensions of economic hardships like material deprivation or economic difficulties/financial strain and their relation to mental health. Some recent studies have however provided evidence of an association between self-reported indicators of current economic difficulties (e.g., difficulties to pay for ordinary bills or pay for rent or lacking cash reserves) with measures of psychological distress and depression [11-13]. However, the correlation between 'income related' (income below a certain poverty line) and other 'non income related' indicators of economic hardships are remarkably low [14,15] and it has been recommended that a household should only be classified as deprived if it has both a low income and other symptoms of deprivation [14].

Furthermore, whatever concepts are used it is clear that problems tend to accumulate among those who already have one or more problems, both material ones and problems affected by lifestyle [16]. While our knowledge of the mechanisms is so far incomplete, it thus seems likely that the harm to health may not only come from economic deprivation, but also from the social and psychological problems of living in poverty. It has also been proposed that the knowledge of having less than others may create feelings of relative depression which in the end also may affect health [17].

In conclusion, many different ways to operationalize and analyse the multifaceted concept of economic hardships have been proposed and the relationship of economic hardships to ill health is intricate.

To date, most of the documented evidence on economic hardships and mental health is restricted to analysing one economic hardship indicator, most often low income, at time. Possible accumulative effects of a combined economic hardship's measure, including both income and non-income related economic hardships measures, on mental health have not been well investigated. Furthermore, gender differences in relation to how economic hardships impact on mental health have hardly been highlighted or evaluated in this field of research.

Poor mental health is an important chronic health problem, representing 4 of 10 of the leading causes of disability [18], with a substantial impact on people's quality of life and societal costs in general [19]. Altogether this makes studies on effects of economic hardships on mental health outcomes of even greater importance, not least as a background for designing more effective policies to reduce health inequalities.

In the present study we aimed at investigating the following; (i) independent associations between multiple measures of economic hardships and mental health problems and (ii) associations of a combined economic hardships measure with mental health problems. We also analysed whether associations differed by gender.

## Methods

### Study population

The analyses were based on the 2009 Swedish National Public Health Survey, which was carried out by Statistics Sweden, in collaboration with a number of various health care regions and districts in Sweden and coordinated by the Swedish National Institute of Public Health. The total study population comprised a randomly selected national sample of 10,373 individuals combined with a randomly selected supplementary sample from four county councils (Halland, Jönköping, Östergötland and Kronoberg) and three municipalities (Gotland, Göteborg and Jönköping) consisting of a total of 51,414 individuals (23,153 men and 28,261 women) aged 16-84 years.

### Data collection

Data was collected within a four-month period during spring (March-June) 2009 and was based on a self-administered questionnaire sent to the respondent's home address. Respondents could choose either to complete the postal questionnaire or to log on to a website and complete a web-based questionnaire. Participants were informed about data linkage with registry data from Statistics Sweden. The response rate was 53.8%. Data from the completed questionnaire were anonymized and controlled for errors, inconsistencies and internal missing data. Missing data were completed by the use of weighting procedures based on related answers from other completed questions, and by the use of weighting procedures based on a calibration method developed by Statistics Sweden [20]. However, weighted procedures were not used in the logistic regression analyses. The present study was approved by the Department of Data Inspection, the Research Ethical Committee at the Swedish National Board of Health and Welfare (20031208) and the Stockholm Regional Ethics Committee (DNR 2005/1146-3 and 2010/1576-32).

### Assessment of variables

#### Outcome variables

A wide range of mental health outcomes were selected in order to get a deeper understanding of the effects of socioeconomic determinants on mental health problems. Mental health problems hence included three measures; (i) psychological distress, (ii) severe anxiety and (iii) use of antidepressant medication.

*Psychological distress *was measured by the 12-item version of the General Health Questionnaire (GHQ-12) [21]. The General Health Questionnaire is a widely used measure of minor psychiatric morbidity that are recent, general and non-psychotic [22] and also predicts more severe mental disorders [22,23]. The GHQ-12 is based on the respondent's assessment of their present state relative to their usual, or normal, state [21]. The 12-item version has been validated in a variety of settings [24-26] and the validity is unlikely to be affected by the language of the questionnaire [22]. A recommended and commonly used cut off point of three or more symptoms was used in this study to indicate psychological distress [22,23].

*Severe anxiety *was based on the question; "*Do you have any symptoms of these; nervousness, uneasiness and anxiety*?" Responses to the question was *"No"*, *"Yes, mild symptoms"*, or *"Yes, severe symptoms"*. Severe anxiety was recorded present if respondents reported having severe symptoms of anxiety or worries. This single item question has been used in previous studies in Sweden where it has been associated with psychiatric disease, ischemic disease and mortality among other things [27].

*Use of antidepressant medication *was based on the question; "*Have you during the past three months used antidepressant medications?" *Alternative answers were "Yes" or "Now". Use of antidepressant medications was recorded as present if respondent answered "Yes". The question has been shown to be a good measure to validate self-reported mental health problems [28].

### Main determinant

#### Economic hardships

Three variables were used to define economic hardships; (i) having a low household income (ii) inability to meet expenses and (iii) lacking cash reserves. Low household income, was based on information on disposable annual household income in the tax register office the year previous to the study. The measure takes into account all income, both wages and benefits, net of taxes and adjusted for price inflation, family size and composition. Household income was categorised as low based on the lowest 20% of the total distribution household income of study participants (less or equal to 182 046 SEK/year, about 19 000 Euros). Low income is a commonly used measure in poverty research showing clear associations with health outcomes [29].

Two complementary indicators of economic hardships capturing self-reported economic difficulties were included; (ii) inability to meet expenses (difficulties in managing current expenditure for food, rent, bills, etc. during the past 12 months) and (iii) lacking cash reserves (not being able to get hold of 15 000 SEK (about 1600 Euros) within a week if needed). These two items have been previously used in Swedish studies to measure subjective economic hardship and have shown clear associations with health outcomes [30,31].

Finnally, we constructed a combined economic hardship measure, including all three indicators, to judge on accumulation of economic hardships. The average inter-item correlation between the three economic hardships measures was low, with a standardised Chronbachs alpha coefficient of 0.48, which would motivate us to refrain from constructing an index based on the three variables. However, we are foremost interested in the combined economic hardships measure as a combination of three separate economic hardships indicators each capturing separate dimensions of the multifaceted construct of economic hardships, and not as an index in statistical terms. Thus, we constructed an index that captures the vulnerabilities from these economic factors, given the social context of Sweden as a welfare state.

The combined economic hardships measure was constructed as follows; the three economic hardships factors were binary recorded and summed up resulting into sums between zero to three. Economic hardships was categorized as 'none' (if the sum was equal to zero), 'mild' (if the sum was equal to one), 'severe' (if the sum was equal to two) and 'very severe' (if the sum was equal to three).

#### Covariates

Age, country of birth, educational attainment, occupational status (register-based data) and employment status, family status, and long-term illness (self-reported data) were adjusted for in the analyses as confounding factors.

*Age *was categorized into 4 age groups: (i) 16-29, (ii) 30-44, (iii) 45-64 and (iv) 65-84 years.

*Country of birth *was categorized as (i) Sweden, (ii) other OECD countries (other Nordic countries, Europe) or (ii) other countries (Africa, Asia, Latin America, Middle East).

*Family status *was based on four categories; (i) living alone without children, (ii) living alone with children, (iii) cohabiting without children or (iv) cohabiting with children in a household. Respondents were categorized as being a lone adult if they reported one of the first two alternatives.

*Educational attainment *was categorized into three levels; (based on the highest examination level passed); (i) low (nine years compulsory school or less), (ii) intermediate (upper secondary school or less), (iii) high (university/college level).

*Occupational status *was categorised as (i) manual workers, (ii) lower non-manual workers, (iii) non-manual workers, (iv) farmers and self-employed or (v) unclassified occupational status.

*Employment status *was categorized as; (i) employed, (ii) other economically inactive (e.g., students, sick-leave absence or maternity leave), (iii) unemployed.

*Long-term illness *is reported to be a confounding factor for mental illness [32], we have therefore adjusted for long-term illness in the multivariate analyses. Long-term illness was based on the "Yes" or "No" answer question; "*Are you suffering from any long-term illness, after effects from accident or other ailment?" *Respondents who answered "Yes" were regarded as suffering from long-term illness.

### Statistical methods

#### Data analyses

Prevalence (%) of mental health problems, economic hardships, demographic, socioeconomic status (educational level and occupational status), and long-term illness variables were calculated, complemented with chi-square test to test for significant gender differences (Table 1). In the logistic regression analyses, we used the regression associations coefficients (standard errors) to obtain OR (95% CI). First, three multiple logistic regression models adjusting for potential confounders (Model 1; age, Model 2; age, country of birth, educational level, financial stress, employment status, family status and long term illness) were run to ascertain independent associations between each of the three economic hardships variables and mental health problems. The three economic hardships variables were also simultaneously adjusted for in Model 2 (along with the other confounders) to evaluate their independent associations (Table 2 and 3). Secondly, we analyzed the association between accumulated economic hardships and mental health problems adjusting for potential confounders (age, country of birth, family status, socioeconomic status variables and long term illness) (Table 3). All analyses were conducted for men and women separately using SAS, version 9.1.3.

**Table 1:** Prevalence (%) of economic hardship (inability to pay for ordinary bills, lack of cash reserves, low household income & a combined economic hardships measure), demographic and socioeconomic variables and mental health problems (GHQ12-psycoholocal distress, severe anxiety & use of antidepressant medication) (the Swedish National Public Health Survey 2009).

	*Men (N = 23,153)*	*Women (N = 28,261)*	P-value*
**Exposure**	% (n)	% (n)	
			
**Inability to pay ordinary bills**			
No	85.7 % (19675)	82.4 % (23048)	
Yes	14.3 % (3275)	17.6 % (4931)	
(Missing)	(203)	(282)	<.0001
**Lack of cash reserves**			
No	83.6 % (19167)	76.7 % (21407)	
Yes	16.4 % (3771)	23.3 % (6512)	
(Missing)	(215)	(342)	<.0001
**Low household income**			
No	83.2 % (19179)	77.5 % (21813)	
Yes	16.8 % (3865)	22.5 % (6349)	
(Missing)	(91)	(99)	<.0001
**Combined economic hardships measure**			
None	67.5 % (15627)	57.8 % (16326)	
Mild	20.9 % (4834)	25.6 % (7225)	
Severe	8.6 % (1996)	12.6 % (3560)	
Very severe	3.0 % (695)	4.1 % (1149)	
(Missing)	(509)	(723)	<.0001
**Background factors**			
**Age (years)**			
16-29	16.2 % (3746)	17.4 % (4906)	
30-44	22.9 % (5304)	24.0 % (6768)	
45-64	36.1 % (8367)	35.8 % (10119)	<.0001
65-84	24.8 % (5736)	22.9 % (6468)	
(Missing)	(0)	(0)	
**Country of birth**			
Sweden	88.5 % (13172)	87.7 % (16074)	
OECD (Other Nordic, Europe, North America)	7.7 % (1151)	8.6 % (1572)	
Other (Asia, Latin America, Africa)	3.8 % (563)	3.8 % (692)	
(Missing)	(8267)	(9923)	<0.0210
**Educational level**			
High	17.9 % (10125)	21.8 % (5611)	
Intermediate	34.1 % (7183)	33 % (8492)	
Low	48.0 % (5772)	45.1 % (11605)	<.0001
(Missing)	(2073)	(2553)	
**Occupational status**			
Non-manual workers	36.8 % (7982)	36.2 % (9470)	
Lower non-manual workers	9.4 % (2034)	17.8 % (4643)	
Workers	41.9 % (9079)	37.8 % (9869)	
Farmers and self-employed	7.4 % (1601)	3.1 % (807)	
Unclassified occupational status	4.6 % (995)	5.2 % (1346)	
(Missing)	(1462)	(2126)	<.0001
**Employment status**			
Employed	57.1 % (12769)	54.1 % (14636)	
Other activities (students, parental leave etc.)	38.4 % (8590)	41.3 % (11174)	
Unemployed	4.4 % (988)	4.7 % (1265)	
(Missing)	(806)	(1186)	<.0001
**Living alone (with or without children)**			
No	69.5 % (15722)	65.8 % (18201)	
Yes	30.5 % (6901)	34.2 % (9442)	
(Missing)	(530)	(618)	<.0001
**Long term illness**			
No	91.0 % (20755)	89.1 % (24723)	
Yes	9.0 % (2048)	10.9 % (3035)	
(Missing)	(350)	(503)	<.0001
**Health outcomes**			
**Psychological distress (GHQ12)**			
No	86.1 % (19858)	81.0 % (22546)	
Yes	13.9 % (3196)	19.0 % (5596)	
(Missing)	(99)	(119)	<.0001
**Severe anxiety**			
No	96.5 % (21911)	94.2 % (26111)	
Yes	3.5 % (805)	5.8 % (1607)	
(Missing)	(437)	(543)	<.0001
**Use of antidepressant medications**			
No	95.1 % (15441)	91.0 % (26671)	
Yes	4.9 % (788)	9.0 % (1810)	
(Missing)	(16924)	(8179)	<.0001

*p-value from chi-square tests

**Table 2:** Age adjusted and multivariate adjusted odds ratios (OR) and 95% confidence intervals (CI) of mental health problems (GHQ12, severe anxiety & use of antidepressant medication) in relation to economic hardship (inability to pay ordinary bills, lack of cash reserves, low household income) Men (the Swedish National Public Health Survey 2009).

	Psychological distress (GHQ12)		Severe anxiety		Use of antidepressant medications	
	**OR (95% CI)**		**OR (95% CI)**		**OR (95% CI)**	
**Men** (*N = 23,153*)						
	**Model 1**	**Model 2**	**Model 1**	**Model 2**	**Model 1**	**Model 2**
**Inability to pay for ordinary bills**						
No (reference)	1.00	1.00	1.00	1.00	1.00	1.00
Yes	3.34 (3.06-3.65)	2.05 (1.76-2.38)	4.92 (4.24-5.72)	2.76 (2.10-3.63)	3.07 (2.80-3.63)	1.90 (1.48-2.43)
**Lack of cash reserves**						
No (reference)	1.00	1.00	1.00	1.00	1.00	1.00
Yes	2.52 (2.31-2.75)	1.38 (1.17-1.62)	4.27 (3.69-4.95)	1.67 (1.26-2.21)	2.85 (2.42-3.34)	1.34 (1.03-1.73)
**Low household income**						
No (reference)	1.00	1.00	1.00	1.00	1.00	1.00
Yes	1.82 (1.67-2.00)	1.14 (0.98-1.33)	2.70 (2.32-3.15)	1.09 (0.83-1.42)	2.64 (2.25-3.09)	1.51 (1.20-1.91)

Model 1 adjusted for age

Model 2 adjusted for age, country of birth, educational level, occupational status, employment status, family status and long term illness and all three economic hardships variables included in the model simultaneously

**Table 3:** Age adjusted and multivariate adjusted odds ratios (OR) and 95% confidence intervals (CI) of mental health problems (GHQ12, severe anxiety & use of antidepressant medication) in relation to economic hardship (inability to pay ordinary bills, lack of cash reserves, low household income) Women (the Swedish National Public Health Survey 2009).

	Psychological distress (GHQ12)		Severe anxiety		Use of antidepressant medications	
	**OR (95% CI)**		**OR (95% CI)**		**OR (95% CI)**	
**Women **(*N *= 28,261)	**Model 1**	**Model 2**	**Model 1**	**Model 2**	**Model 1**	**Model 2**
**Inability to pay for ordinary bills**						
No (reference)	1.00	1.00	1.00	1.00	1.00	1.00
Yes	2.56 (2.38-2.74)	1.81 (1.61-2.04)	3.56 (3.19-3.97)	1.85 (1.52-2.24)	2.67 (2.39-2.99)	1.55 (1.30-1.84)
**Lack of cash reserves**						
No (reference)	1.00	1.00	1.00	1.00	1.00	1.00
Yes	2.01 (1.88-2.15)	1.34 (1.20-1.51)	2.94 (2.65-3.27)	1.63 (1.34-1.98)	2.01 (1.88-2.32)	1.21 (1.02-1.43)
**Low household income**						
No (reference)	1.00	1.00	1.00	1.00	1.00	1.00
Yes	1.44 (1.34-1.55)	1.14 (0.99-1.28)	1.89 (1.69-2.12)	1.09 (0.91-1.31)	1.81 (1.62-2.03)	1.18 (0.99-1.39)

Model 1 adjusted for age

Model 2 adjusted for age, country of birth, educational level, occupational status, employment status, family status and long term illness and all three economic hardships variables included in the model simultaneously

## Results

The characteristics of the sample population are summarised in Table 1. Approximately one fifth of the women and one sixth of the men reported inability to pay for ordinary bills. Analysis of the combined economic hardships measure revealed that 17% of the women suffered from severe or very severe levels of economic hardships compared to 12% of the men. 19% of the women and 14% of the men reported that they suffered from psychological distress, 6% of women and 4% of men reported that they suffered from severe anxiety and 5% of the men and 9% of the women reported use of antidepressant medications.

In Table 2 and 3 we show associations between each of the three economic hardship variables and mental health problems simultaneously adjusting for potential confounders and all the three economic hardship variables. We found that the association between the two self-reported economic hardship variables; (i) inability to pay for ordinary bills and (ii) lack of cash reserves and all indicators of mental health problems were consistently statistically significant for both men and women. For example, the multivariate adjusted regression analysis (Model 2) show that women with inability to pay for ordinary bills had an increased risk of 1.81 for psychological distress; 1.85 for severe anxiety and 1.55 for use of antidepressant medications compared with women with no inability to pay for ordinary bills. The corresponding figures for men were 2.05 for psychological distress; 2.76 for severe anxiety and 1.90 for use of antidepressant medications. Conversely, associations between low income and all indicators of mental health problems were consistently not statistically significant for both men and women (with the exception of a 50% higher risk for use of antidepressant medications among men with a low income compared to men with a high income).

In Table 4 independent associations between our combined economic hardships measure and mental health problems adjusting for potential confounders (age, country of birth, family status, occupational status, employment status, educational level and long term illness) are presented. Model 1 (adjusted for age) show that compared with women without economic hardship, those women with very severe economic hardship had a three-fold increase in odds for psychological distress and a six-fold risk of severe anxiety and a four-fold use of antidepressant medications. Among men with very severe economic hardships we found a five-fold increase in odds for psychological distress and a ten-fold risk of severe anxiety and a six-fold use of antidepressant medications, compared with men without economic hardships.

**Table 4:** Age adjusted and multivariate adjusted odds ratios (OR) and 95% confidence intervals (CI) of mental health problems (GHQ12, severe anxiety & use of antidepressant medication) in relation to the level of economic hardships (the Swedish National Public Health Survey 2009).

	*Psychological distress (GHQ12)*		*Severe anxiety *		*Use of antidepressant medications*	
	**OR (95% CI)**		**OR (95% CI)**		**OR (95% CI)**	
**Men **(*N = *23,153)	**Model 1**	**Model 2**	**Model 1**	**Model 2**	**Model 1**	**Model 2**
						
**Combined economic**						
**hardships measure**						
None (reference)	1.00	1.00	1.00	1.00	1.00	1.00
Mild	1.73 (1.58-1.91)	1.52 (1.32-1.76)	2.45 (2.03-2.96)	1.53 (1.12-2.08)	1.91 (1.59-2.29)	1.55 (1.21-1.97)
Severe	3.52 (3.15-3.93)	2.55 (2.14-3.05)	6.40 (5.30-7.73)	3.87 (2.85-5.25)	3.67 (2.99-4.52)	2.74 (2.09-3.61)
Very severe	5.40 (4.59-4.34)	3.07 (2.35-4.01)	10.9 (8.63-13.77)	4.83 (3.25-7.16)	6.94 (5.36-8.99)	3.58 (2.47-5.20)
						
**Women **(*N *= 28,261)						
						
**Combined economic**						
**hardships measure**						
None (reference)	1.00	1.00	1.00	1.00	1.00	
Mild	1.50 (1.40-1.62)	1.49 (1.33-1.66)	1.83 (1.60-2.10)	1.53 (1.24-1.88)	1.50 (1.33-1.70)	1.00
Severe	2.57 (2.36-2.79)	2.21 (1.94-2.53)	3.89 (3.39-4.45)	2.67 (2.15-3.32)	2.78 (2.43-3.19)	1.28 (1.09-1.50)
Very severe	3.72 (3.28-4.23)	2.63 (2.16-3.21)	6.82 (5.74-8.11)	3.01 (2.27-4.04)	4.22 (3.50-4.09)	1.78 (1.47-2.15)

Model 1 adjusted for age

Model 2 adjusted for age, country of birth, educational level, occupational status, employment status, family status and long term illness

The odds ratios were considerably reduced after further adjustment for country of birth, educational level, occupational status, employment status, family status and long term illness (Model 2), reducing the odds ratios for women for the "very severe economic hardship" category to 2.6 for psychological distress, 3.0 for severe anxiety and 2.1 for use of antidepressant medications. The corresponding data for men were 3.1 for psychological distress, 4.8 for severe anxiety and 3.6 for use of antidepressant medications. Furthermore, the likelihood of mental health problems differed significantly in a graded fashion in relation to levels of economic hardship. Finally, we rerun the analyses using a different cut-off for psychological distress (GHQ-12 > 3). This did however only marginally alter our results.

## Discussion

First, the results from the present study indicate that self-reported 'current economic difficulties' (inability to pay for ordinary bills and lack of cash reserves), was significantly associated with both women's and men's mental health problems (psychological distress (GHQ-12), severe anxiety and use of antidepressant medication) after mutual adjustments for potential confounders and low income. Conversely we did not find any statistical significant association between low income and mental health problems, neither for women nor for men (with the exception of a 50% higher risk for use of antidepressant medications among men with a low income compared to men with a high income).

Our first main finding reconfirms the results from some recent studies, e.g., in a previous cross-sectional study comparing cohorts of middle-aged Finnish and British employee's self-reported economic difficulties (i.e. difficulties in paying bills and affording food and clothes that the family needs) were associated with common mental disorders (GHQ-12). Such associations were not found for other socioeconomic circumstances, e.g. income (belonging to the lowest income quartile) [12]. In addition, while a longitudinal study of the Canadian National Population Health Survey found financial strain (not enough money to buy necessities) and low educational level to be associated with an increased risk of major depression among the working sample population, no such association was found for low household income [33]. In the Swedish context, the results are also (at least in part) in accordance with the results from a longitudinal study which have shown that exposure to long-term (several episodes during a 16-year period) self-reported economic difficulties was significantly associated with mental health problems (anxiety or worries) for women, while low income was not [11].

However, results from previous studies are not consistent. A vast number of previous studies have, in contrast to our study and some of the studies mentioned above, found evident associations between measures of low income and mental health outcomes [2,6,9]. Then again, other studies have concluded that income, *per se*, is a rather weak predictor of psychological distress and depression [34-36].

The conflicting results may be explained by different samples and methods used, as well as the national and cultural context [10,37]. It has for instance been suggested that the egalitarian socioeconomic policies in the Nordic countries may have reduced the effect of income on health.

Second, we found significant associations of the combined economic hardships measure with all mental health outcomes (psychological distress (GHQ-12), severe anxiety and use of antidepressant medication), which were stable and strong even after adjustments for potential confounders including education and occupation. Furthermore, the likelihood of mental health problems differed significantly in a graded fashion in relation to levels of economic hardships. Hence, our second main finding indicates accumulative effects of economic hardships on mental health outcomes. This issue, has not been widely examined thus results from this paper being an important contribution. Most of the previous studies have either analysed economic hardships variables separately (as presented earlier) or combined them into deprivation or socioeconomic disadvantage indices together with a wide range of other 'welfare problems' [35,38,39].

Even if the differentiation between possible etiologic mechanisms was beyond the scope of this paper several mechanisms might be involved. First, economic hardships could be regarded as a material factor restraining individual's choices due to a pure financial factor. For instance, persons with higher-income jobs may afford greater social prestige and better psychosocial and physical working conditions, which in turn may be protective against depression. A high income also most certainly means greater financial resources to treat depression. It could also be regarded as a psychosocial stressor, affecting health directly by means of psychobiological pathways or indirectly by means of coping processes involving health related behaviours. Hence, it is also possible that worries over the financial situation may either bring about or deteriorate episodes of depression.

Our results highlight the importance of current economic difficulties to mental health problems. 'Current economic difficulties' may thus represent a domain of economic hardships which is not fully captured by the conventional income measures [40]. Furthermore, self-reported measures of current economic difficulties might even possibly be stronger predictors of ill health than low income as they represent more immediate and accumulated influences, which may be more closely related to health than more distal ones [41]. A paper by Weich and Lewis has even suggested that self-reported economic difficulties (labelled as financial strain) is a better independent factor of future psychiatric morbidity than poverty and unemployment [13].

However, the accumulative effects observed in the present study indicate that both 'being poor' and 'feeling poor' together are factors that combined have aggravating effects on mental health. It is hence possibly true that the three economic hardships indicators analysed in the present study all capture separate dimensions of the multifaceted construct of economic hardships (which is supported by the low intra-correlation between the three variables) and together enhance each other and all capture different pieces of the puzzle. This is in line with Mowafi & Khawaja, who have stated that; *"Far from simple, poverty is multidimensional in its symptoms, multivariate in its causes, dynamic in its trajectory, and quite complex in its relation to health"*[42]*(p.1)*. Future research further clarifying which aspects of economic hardships is most important in driving mental health outcomes would contribute to our understanding of economic hardships as a whole.

We also aimed at analysing gender differences in this study. While we found small but significant differences in the prevalence of economic hardships and mental health problems by gender, with higher prevalence's reported for women, the odds ratios of mental health problems in relation to economic hardship (all measures) where quite similar and in some cases somewhat higher for men than for women. We further complemented our analyses by quantifying a possible interaction effect between economic hardships and gender on mental health outcomes (GHQ-12, severe anxiety & use of antidepressant medication) by calculating a synergy index. However, we found no sign of interaction, rather independence of effects between genders (not shown in table).

Some previous studies are in accordance with these finding [12,34,41,43,44], while some other studies show differential associations for women and men [9,27,45,46]. Thus, gender differences in relations to economic hardships and mental health need further scrutiny.

## Strengths and limitations

First, we had the advantage of analysing a large dataset representative of the general population in Sweden. However, the data are cross sectional which did not allow us to make firm conclusions about causality of the observed associations. Nevertheless, previous longitudinal studies have demonstrated that the main direction of causation runs from income and economic difficulties to poor health outcomes [13,33,36,47].

Even so, a possible reverse causality has been shown for depression and/or related symptoms with reduced educational attainment [48] and reduced work productivity [49], which may consequently have negative effects on income and economic conditions. We adjusted for long-term illness but this did not alter the observed relationship between economic hardships and mental health outcomes. Additionally, we run further analysis while stratifying on long term illness and we still observed significant associations between economic hardships and poor mental health. Our results are consistent with a previous study by Lynch et al [6] which suggests that the overall impact of physical disease on the socioeconomic status-mental health (depression) relation is slight.

It is plausible that economic hardships may be a marker of some other factors that cause poor health as indicated by previous studies on associations between psychological distress and low social class [10,50]. Further still, unemployment, which is associated with socioeconomic indicators, has been shown to be a strong risk factor for common mental disorders [51]. Thus unemployment is regarded as substantial for the reversed causation. We adjusted for individual level socioeconomic indicators, including employment status and we still found significant graded associations between economic hardships and poor mental health problems.

We did not have the possibility to control for other individual psychological factors, and this may have confounded the observed association between economic hardships and poor mental health outcomes. Negative affectivity is demonstrated to influence the disposition to respond negatively in surveys [52] and thus may have influenced our results. However, Laaksonen et. al did not find that a personality trait such as negative affectivity influenced the observed associations between socioeconomic circumstances and common mental disorders in their study of middle-aged Finnish and British employees [12].

The cross-sectional nature of the present study did not allow us to address the issue of time lag. Even if we hypothesize that the relationship runs from economic hardships to poor health, it is still plausible that variations in induction time for economic hardships may impact on mental health in different ways [2].

Possible measurement errors in the explanatory variables also need to be given some consideration. However, data on income level were obtained from the register data which is reliable and objective. Other measures are self-reported data which may inhibit a possibility of reporting bias [53]. Still, the self-reports are based on validated instruments which are commonly used in other studies and demonstrated to be associated with poor health outcomes and mortality [30,31].

In order to increase the robustness of the results in this national survey, we analysed three measures of mental health problems. However, the precision of these measures may have been limited by use of a general household questionnaire rather than a standardised clinical interview. Associations between economic hardships and common mental disorders are generally stronger in studies using standardised clinical interviews [54]. On the other hand the use of well-structured and validated instruments measuring different aspects of mental health and control for long-term illness may have minimized this problem.

It is possible that non responders differed from responders. The non-response rate of 46.2% is problematic. However, missing data were completed by the use of weighting procedures based on related answers from other completed questions, and by the use of weighting procedures based on calibration method developed by Statistics Sweden [20,55]. Nevertheless, it is known in national surveys that non-responders are usually over-represented by persons with low socioeconomic position and those with mental health disorders. Thus, results represented in this study may demonstrate an underestimation of the true associations between economic hardships and mental health disorders.

## Conclusions

The results from the present study show that indicators of self-reported economic difficulties are more strongly associated with poor mental health outcomes than the more conventional indicator, low income level. Furthermore, the likelihood of mental health problems differed significantly in a graded fashion in relation to levels of economic hardships. The associations were sustained even after adjustment for education and occupation. The results were also true for both women and men, with very small gender differences. Findings in the present study demonstrate a new finding that economic hardships and not low income per se contribute significantly to mental health disorders. The fact that low income in itself is not generally associated with poor mental health provides substantial implications on policy alternatives. Policies that target poverty reduction with an aim to enhance health should not only focus on the level of income, they should also focus on how people in poverty can manage their daily basic needs that can increase their capabilities, in the same way as Amartya Sen demonstrates [56,57].

## Abbreviations

GHQ12: General Health Questionnaire, 12 item version; OR: Odds Ratio

## Competing interests

The authors declare that they have no competing interests.

## Authors' contributions

JA participated in the design of the study, was responsible for preparation of the dataset, performed the statistical analysis and drafted the manuscript. SW conceived of the study, and participated in its design and coordination and helped to draft the manuscript. Both authors read and approved the final manuscript.

## Pre-publication history

The pre-publication history for this paper can be accessed here:

http://www.biomedcentral.com/1471-2458/11/788/prepub
